# Applied anatomy of pelvic lymph nodes and its clinical significance for prostate cancer:a single-center cadaveric study

**DOI:** 10.1186/s12885-020-06833-1

**Published:** 2020-04-16

**Authors:** Jia-Jun Chen, Zai-Sheng Zhu, Yi-Yi Zhu, Hong-Qi Shi

**Affiliations:** 1 Department of Urology, Jinhua Municipal Central Hospital, JingHua, China; 2 Zhejiang University School of Medicine, HangZhou, China; 3 Department of Urology, ShaoXing People’s Hosptial, ShaoXing, China; 4 Jinhua Municipal Central Hospital, Department of Urology, No. 365 Renmin East Road, Jinhua City, 321000 Zhejiang Province China; 5 Jinhua Municipal Central Hospital, Department of Pathology, JingHua, China

**Keywords:** Anatomy, Lymph node count, Pelvic lymph node dissection, Prostate cancer

## Abstract

**Background:**

Pelvic lymph node dissection (PLND) is one of the most important steps in radical prostatectomy (RP). Not only can PLND provide accurate clinical staging to guide treatment after prostatectomy but PLND can also improve the prognosis of patients by eradicating micro-metastases. However, reports of the number of pelvic lymph nodes have generally come from incomplete dissection during surgery, there is no anatomic study that assesses the number and variability of lymph nodes. Our objective is to assess the utility of adopting the lymph node count as a metric of surgical quality for the extent of lymph node dissection during RP for prostate cancer by conducting a dissection study of pelvic lymph nodes in adult male cadavers.

**Methods:**

All 30 adult male cadavers underwent pelvic lymph node dissection (PLND), and the lymph nodes in each of the 9 dissection zones were enumerated and analyzed.

**Results:**

A total of 1267 lymph nodes were obtained. The number of lymph nodes obtained by limited PLND was 4–22 (14.1 ± 4.5), the number obtained by standard PLND was 16–35 (25.9 ± 5.6), the number obtained by extended PLND was 17–44 (30.0 ± 7.0), and the number obtained by super-extended PLDN was 24–60 (42.2 ± 9.7).

**Conclusions:**

There are substantial inter-individual differences in the number of lymph nodes in the pelvic cavity. These results have demonstrated the rationality and feasibility of adopting lymph node count as a surrogate for evaluating the utility of PLND in radical prostatectomy, but these results need to be further explored.

## Background

Pelvic lymph node dissection (PLND) is one of the most important steps in radical prostatectomy (RP). Not only can PLND provide accurate clinical staging to guide treatment after prostatectomy but PLND can also improve the prognosis of patients by eradicating micro-metastases [1–3]. However, there is no anatomic study that assesses the number and variability of lymph nodes. In addition, the scope of PLND, the indications for PLND and the number of lymph nodes that should be resected remain inconclusive [4, 5]. The purpose of this study was to investigate the utility of adopting the lymph node count as a criterion to evaluate PLND during RP by dissecting and observing 30 adult male human cadaver specimens and counting the lymph nodes.

## Methods

The subjects consisted of 30 male cadavers donated to the Department of Anatomy, Jinhua Polytechnic, for medical teaching and research. The cadavers aged ≥18 years with no history of pelvic (bladder, prostate, etc.) malignancies, lymphoma, pelvic irradiation, or pelvic surgery were eligible for the present study. The cadavers were deidentified, and the available information included age and cause of death. The cause of death was categorized according to the following 5 groups: cardiovascular causes, chronic obstructive pulmonary disease, traumatic brain injury, organic brain dysfunction and lung cancer. The Jinhua Central Hospital Ethics Review Board approved the study.

### Anatomy and observation of the pelvic lymph nodes in the cadaveric specimens

**Fig. 1:**
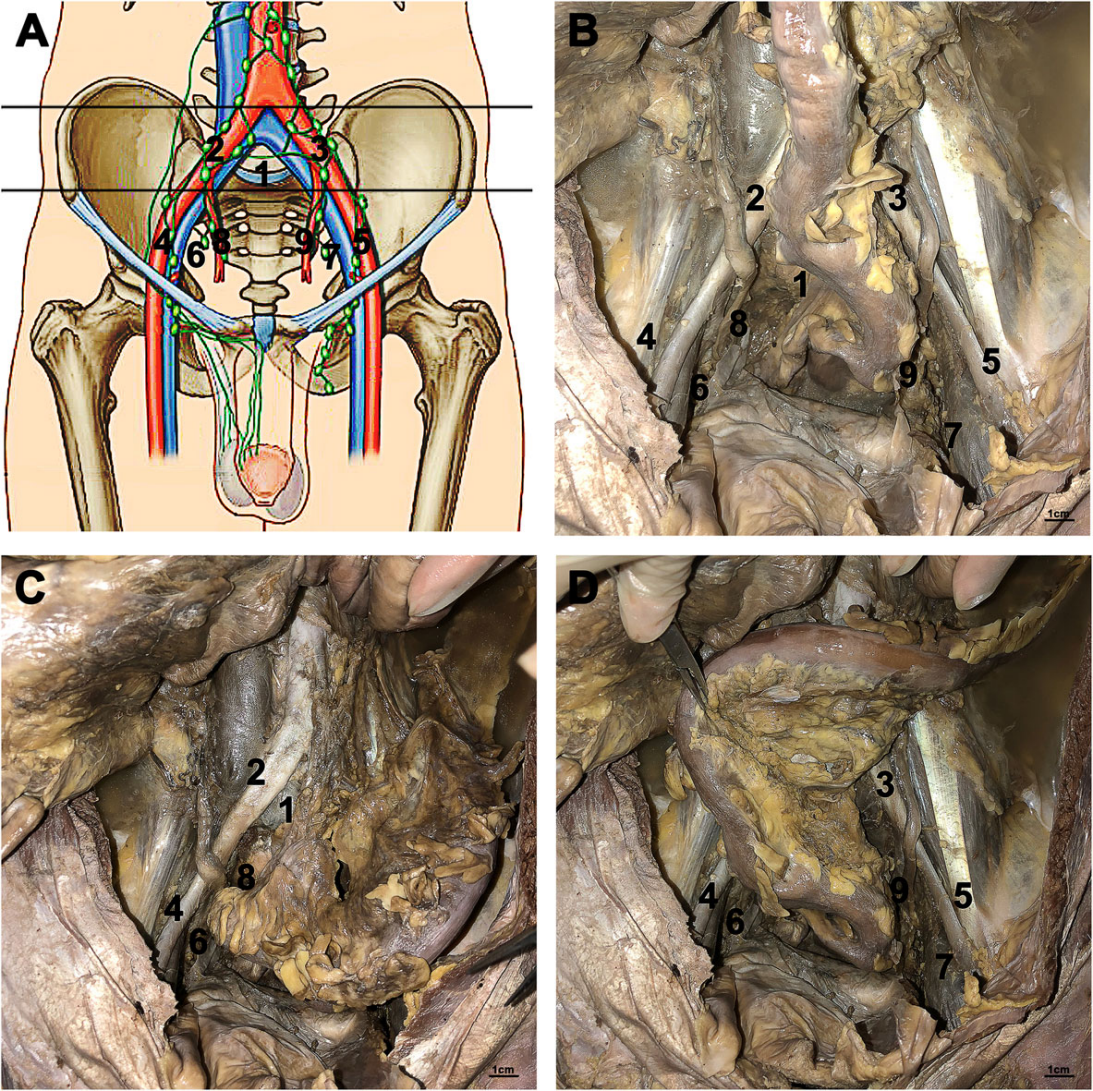
Distribution of pelvic lymph nodes 1: presacral nodes; 2: right common iliac nodes; 3: left common iliac nodes; 4: right external iliac nodes; 5: left external iliac nodes; 6: right obturator nodes; 7: left obturator nodes; 8: right internal iliac nodes; 9: left internal iliac nodes

All 30 adult male cadavers underwent pelvic lymph node dissection (PLND) by the same anatomists (Zaisheng Zhu, Jiajun Chen.), and all nodes were counted and recorded by a single pathologist (Hongqi Shi). The boundaries of the lymph node dissection were as follows: the cephalic boundary was the bifurcation of the abdominal aorta; the caudal boundary was the circumflex iliac vein and Cooper ligament; the external boundary was the genitofemoral nerve; and the posterior boundary was the internal iliac artery (Fig. 1).

We used a dissection template that included the following 9 zones: 1, Presacral nodes: below the bifurcation of the abdominal aorta and inferior vena cava, in the triangle between the left and right common iliac vessels. All lymph nodes and fibrous adipose tissue in front of the L5 vertebral body and sacroiliac joint were removed; 2 and 3, Common iliac nodes: fibrous lymphatic adipose tissue around the common iliac artery and vein, including nodes in the anterior ischial region (Marcille’s fossa), was removed; 4 and 5, External iliac nodes: from the upper edge of the external iliac artery to the lower edge of the external iliac vein and from the bifurcation of the common iliac artery to the inguinal canal, all fibrous lymphatic adipose tissue including the croquet nodes was removed; 6 and 7, Obturator nodes: the superior edge was the external iliac vein, and the inferior edge was the obturator nerve; the head side was the bifurcation of the common iliac vein, and the tail side was the inferior edge of the external iliac vein and the pubic bone. All fibrous tissue within this zone was removed; 8 and 9, Internal iliac nodes: the upper margin was the obturator nerve, and the lower margin was the lateral margin of the prostatic nerve vascular bundle; from the ureter to the obturator, all lymph nodes and fibrous adipose tissue between the side of the iliopsoas muscle and the internal iliac artery, including all branches of the internal iliac artery to the bifurcation of the common iliac artery, were removed.

The tissue samples from each dissection zone were packaged separately and stored in 10% formalin solution until evaluation. Lymph nodes in adipose connective tissue are counted as follows: cut the tissue at intervals of 0.5 cm (do not cut completely, but maintain a continuous sequence between adjacent tissue slices), then use magnifying glass to distinguish and feel with fingers. Adipose lobules and small lymph node are not easy to distinguish in appearance sometimes, press with finger slightly, adipose lobule is easy to be crushed, have oily and lubricious feeling, while lymph node has capsule, not easy to be crushed. All lymph nodes were examined under a microscope.

Based on the 2017 EAU guidelines [6], the dissection scope was divided into the following four levels: limited PLND (obturator nodes), standard PLND (s-PLND) (obturator nodes + external iliac nodes), e-PLND (s-PLND+ internal iliac nodes), and super-extended PLND (e-PLND+ common iliac nodes).

### Statistical methods

Data were processed using IBM SPSS Statistics 23.0. The number of lymph nodes in each dissection zone was counted and analyzed. The results are presented as the mean ± standard deviation (SD) for normally distributed data. The coefficient of variation (CV) in each dissection level and each region was calculated to compare the degree of dispersion between groups. Paired t-tests were used to compare the lymph node counts in the limited PLND and e-PLND.

## Results

**Fig. 2:**
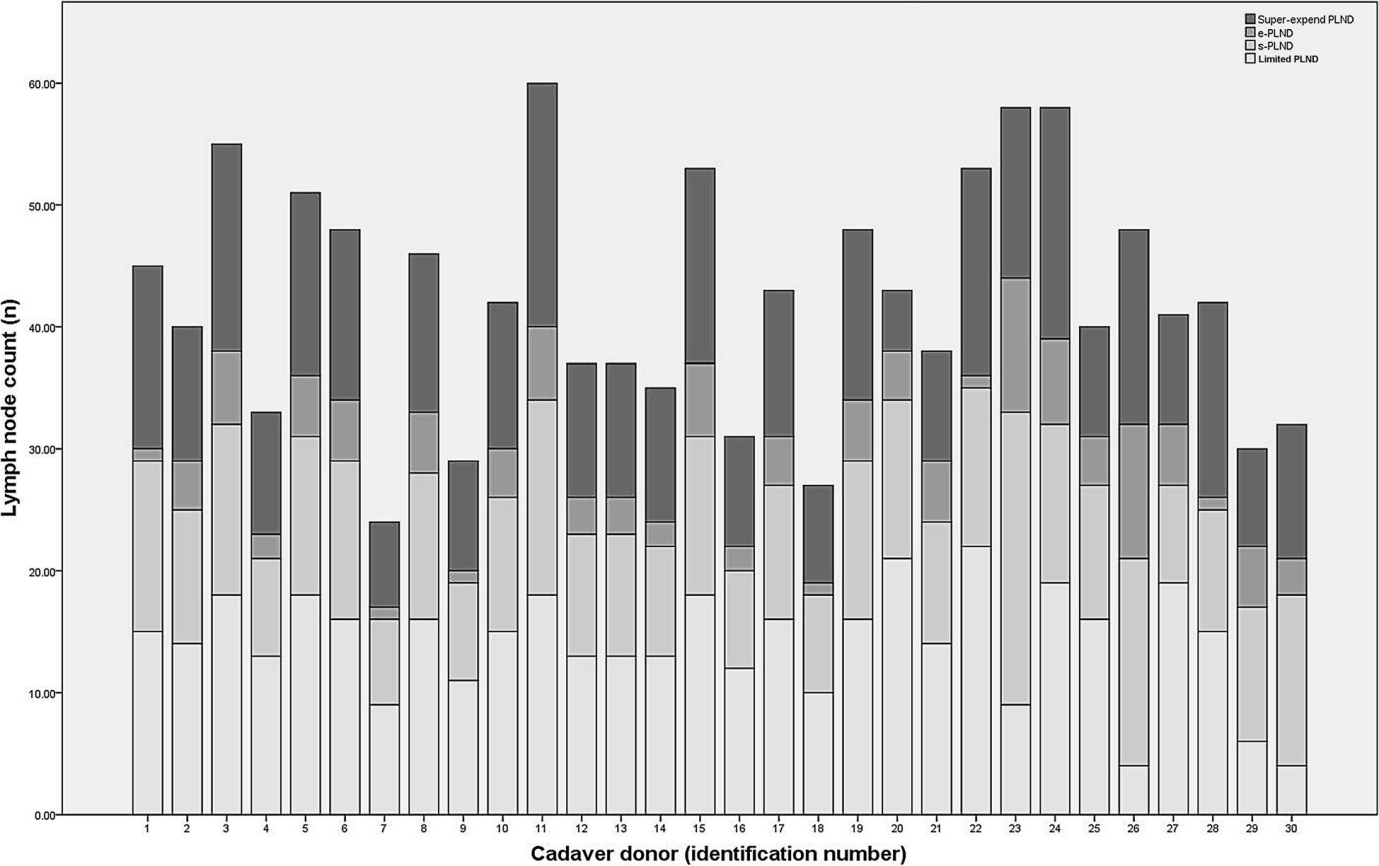
Inter-individual variability in the node count within different dissection scope leval

A total of 30 cadavers met the criteria and underwent dissection. The mean age at death was 71.9 ± 10.0 years, and the causes of death were cardiovascular causes for 12, chronic obstructive pulmonary disease for 5, traumatic brain injury for 6, organic brain dysfunction for 5 and lung cancer for 2 cadavers. A total of 1267 lymph nodes were obtained. The distribution and variability of lymph node counts within each dissection region is illustrated in Table 1. The marked inter-individual variability in the node count within the different dissection scope levels is shown in Fig. 2.

**Table 1:** Distribution of pelvic lymph nodes in 30 adult male cadavers

Region			No.(x¯ ± s)	CV(%)
1	Presacral		88 (2.9 ± 2.5)	84.4
2	Common iliac	Left	144 (4.8 ± 1.7)	34.8
3		Right	136 (4.5 ± 1.4)	31.1
4	External iliac	Left	180 (6.0 ± 1.8)	29.4
5		Right	173 (5.8 ± 2.1)	37.2
6	Obturator	Left	201 (6.7 ± 2.2)	33.5
7		Right	222 (7.4 ± 3.2)	43.3
8	Internal iliac	Left	60 (2.0 ± 1.2)	61.6
9		Right	63 (2.1 ± 1.6)	78.4

**Fig. 3:**
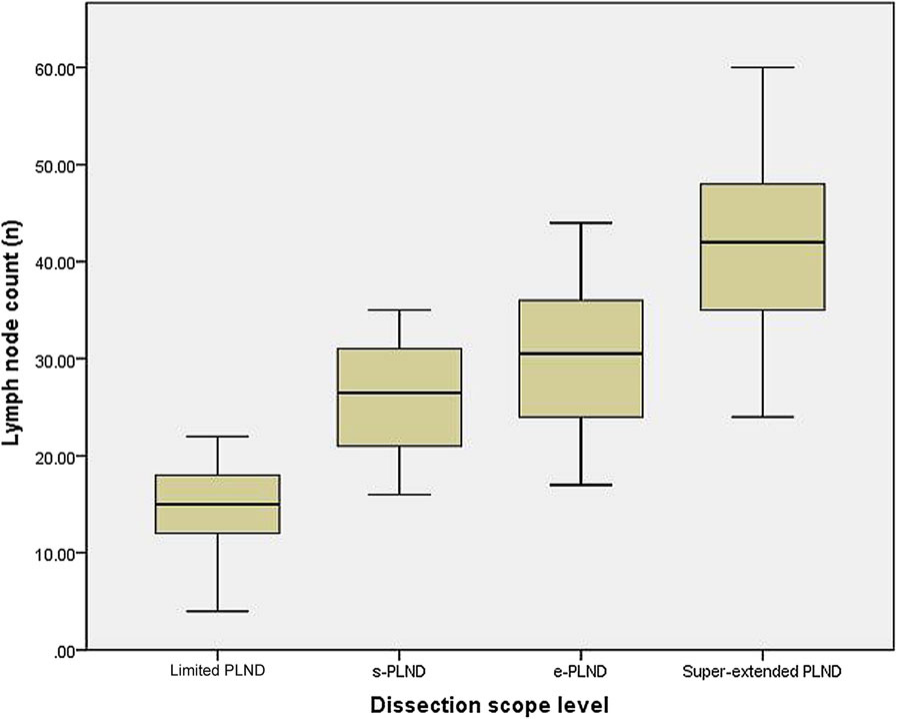
Box plot comparing diffrernt PLND dissection scope leval

The number of lymph nodes obtained by limited PLND was 4–22 (14.1 ± 4.5, CV 32.2%), by s-PLND was 16–35 (25.9 ± 5.6, CV 21.5%), by e-PLND was 17–44 (30.0 ± 7.0, CV 23.3%) and by super-extended PLDN was 24–60 (42.2 ± 9.7, CV 23.1%) (Fig. 3). A total of 15.9 ± 5.6 additional nodes were obtained using e-PLND instead of limited PLND (*P* < 0.001), with the dissection from the external iliac area yielding 11.8 ± 3.4 nodes and the internal iliac region yielding 4.1 ± 2.6 nodes.

## Discussion

PLND is an important step in RP. According to the 2017 EAU guidelines, extended PLND (e-PLND) is indicated for patients with prostate cancer whose positive rate of lymph node biopsy is more than 5%, as estimated by the preoperative risk scale [6]. PLND before RP has been considered to be of great significance in the diagnosis of prostate cancer, and the therapeutic value of PLND has been gradually valued in recent years [7, 8]. PLND can not only provide accurate clinical staging but can also enable the removal of microcarcinomas, which is beneficial for improving the prognosis of patients [9]. Some studies have reported that radical prostatectomy can improve the long-term outcomes for some patients with limited lymph node metastasis [8, 10, 11]. However, the scope of PLND, the indications for PLND and the number of lymph nodes that should be resected remain inconclusive [4, 5].

### The number of pelvic lymph nodes

Lymph node count is the most commonly used method to evaluate the extent of PLND. Canessa et al. cleared pelvic lymph nodes below the bifurcation of iliac vessels in 16 cadavers and obtained a mean of 28.6 (16–46) nodes [12]. In our e-PLND, the mean number of nodes obtained was 30.0 (17–44), which was similar to that reported by Canessa but more than that reported by most clinical operations [13, 14]. This may be because the number of lymph nodes removed during clinical operations can be affected by many factors, including the scope of dissection, the amount of tissue obtained, and the surgeon’s experience [12]. Of course, this difference may also be caused by racial differences and individual differences between patients. In this study, all pelvic lymph nodes and fibrous adipose tissues could be completely removed by autopsy without paying attention to the operation time and complications, and the number, anatomical distribution and variation of lymph nodes was accurately evaluated.

Fleischmann et al. reported that the number of lymph nodes was variable. The number of lymph nodes obtained by e-PLND ranged from 10 to 43 [15]. Even with the same anatomical range, the number of lymph nodes obtained by different doctors is different. For example, the total number of pelvic lymph nodes (super-extended PLND+ peri-aortic lymph nodes) has been reported to be 50.6 ± 14 [16], 43.1 ± 16 [17] and 37 (27–49) [18]. The mean number of lymph nodes obtained by limited PLND, s-PLND, e-PLND and super-extended PLDN was 14, 26, 30 and 42, respectively, which indicates that the number of lymph nodes is closely related to the anatomical area of the pelvis, and its distribution does not decrease with the increased distance from the prostate and other pelvic organs. The CV was 32.2, 21.5, 23.3, and 23.1% for limited PLND, s-PLND, e-PLND, and super-extended PLND, respectively, which further indicated that there were significant individual differences in the number of pelvic lymph nodes. Therefore, we consider that in PLND, we should not only pay attention to the number of pelvic lymph nodes but also to the scope of their dissection.

### The scope of PLND

As early as 2007, Heidenreich et al. [19] found that more lymph nodes could be obtained and staged more accurately with e-PLND than with limited PLND; e-PLND could obtain 21–28 lymph nodes, while limited PLND could obtain 10–11, and the positive rates were 26 and 12%, respectively. They suggested that PLND should include the internal iliac, external iliac, common iliac, obturator and presacral lymph nodes [20].

Some researchers have proposed the concept of early lymph node metastasis (sentinel lymph node) of prostate cancer, in which the metastases mainly distribute in the obturator, external and internal iliac regions. Bader et al. [21] reported 365 patients who underwent RP and e-PLND, 25% of whom had confirmed lymph node involvement after operation, while approximately 20% of these patients only had metastases that invaded the internal iliac nodes. A. Briganti et al. [22] analyzed 1636 cases of PLND and found that approximately 50% of the positive lymph nodes were detected outside the obturator node area. Moreover, Gandaglia et al. [23] found that 62.5, 55.2 and 47% of the positive lymph nodes were located in the obturator, internal iliac and external iliac regions, respectively, while only 5.3 and 2.5% of the lymph nodes in the common iliac and presacral regions were positive, respectively.

Mattei A et al. [24] reported that e-PLND could remove 75% of the lymph nodes with potential metastasis risk. Joniau S et al. [25] reported that e-PLDN+ presacral nodes could remove 97% of the lymph nodes and 88% of the metastatic lymph nodes. Our group has previously reported data on 103 patients who underwent RP+ e-PLND [26], and we found the following lymph node metastasis rates: internal iliac nodes, 59% (13/22); obturator nodes, 50% (11/22); external iliac nodes, 36% (8/22): presacral nodes, 14% (3/22); and common iliac nodes, 5% (1/22) (*P* < 0.05). The lymph node metastasis density was 28% (21/74), 37% (19/53), 25% (8/32), 33% (3/9) and 20% (1/5) for the internal iliac, obturator, external iliac, presacral, and common iliac nodes, respectively (*P* > 0.05). We propose that the sentinel lymph nodes of prostate cancer, including the obturator, external and internal iliac nodes that have high metastasis rates and densities, need to be removed during PLND. If suspicious lymph nodes are found in the presacral region, they should also be removed, but iliac area does not need regular dissection.

### The relationship between the number of lymph nodes resected and prognosis

Heidenreich et al. [19] reported that e-PLND could significantly reduce the cancer-specific mortality (CSM) of prostate cancer (23% reduction in N + and 15% reduction in N0). Many researchers have tried to reduce the number of resected nodes to reduce postoperative complications while ensuring tumor control. JI JD et al. [27] reported data on 360 patients with localized prostate cancer who underwent open RP. A comparison of the progression-free survival after s-PLND (obturator and external iliac nodes) to that after e-PLND (obturator, internal iliac, external iliac and common iliac nodes) revealed that the 5-year progression-free survival rates after s-PLND and e-PLND were 90.1 and 91.3% in the low-risk group, respectively. There was no significant difference between the survival rates. In contrast, there was a significant difference in the intermediate risk group (73.1% vs. 85.7%, *P* = 0.042) and in the high-risk group (51.1% vs. 71.4%, *P* = 0.036). Abdollah et al. [28] reported data on 315 cases of lymph node metastasis. They found that the ratio between the number of resected lymph nodes and the 10-year survival rates without CSM was 8:74.7%, 17:85.9%, 26:92.4%, 36:96% and 45:98%. CSM was significantly reduced when the number of resected lymph nodes was 14 or more.

However, there are different views on the relationship between the number of lymph nodes resected and prognosis, such as that from the report from the 2017 meeting of the American Society of Clinical Oncology. A phase III randomized controlled trial in Brazil showed that e-PLND can improve the accuracy of clinical staging, but the short-term follow-up did not demonstrate oncological benefits. There was no benefit in biochemical recurrence (BCR), radiotherapy efficacy, androgen-deprivation therapy (ADT) efficacy, bone metastasis or mortality, and e-PLND significantly increased operative time, intraoperative bleeding volume, length of hospital stay and incidence of postoperative complications [29]. We believe that this is a high-level evidence-based medical research study, but we cannot deny the findings of all previous studies. After all, research data from real clinical practices are also convincing. The results of that trial need to be followed up, and further prospective, randomized, controlled, multicenter studies are needed to confirm their findings.

Limitations: The Limitations of this study include the following: (1) approximately 90% of the patients who undergo RP are 70 years or younger [30], but the mean age of the specimens was 71.9 years; (2) the past histories of the cadavers in the study group were not detailed enough. It is not clear whether they suffered from chronic pelvic organ disorders, prostatitis, etc.; and (3) the number of autopsy specimens was not large enough. We hope to further accumulate cases to obtain more objective and accurate results to contribute to the conclusions on PLND.

## Conclusion

We have demonstrated that an average of nearly 30 lymph nodes can be expected from e-PLND compared with an average of approximately 14 nodes from limited PLND. However, there are substantial inter-individual differences in the number of lymph nodes in the pelvic cavity, and we found lymph node counts ranging from 24 to 60 nodes with super-extended PLND (CV 23.1%). These results have demonstrated the rationality and feasibility of adopting lymph node count as a surrogate for evaluating the utility of PLND in radical prostatectomy, but these results need to be further explored.

## Data Availability

Reasonable requests for data and materials will be considered and should be made in writing to the corresponding author.

## Abbreviations

CV: Coefficient of variation
e-PLND: Extended PLND
PLND: Pelvic lymph node dissection
RP: Radical prostatectomy
SD: Standard deviation
s-PLND: Standard PLND
